# Supplementary material for: Microbial diversity of extreme habitats in human homes
Source: PeerJ. 2016 Sep 13;4:e2376. doi: 10.7717/peerj.2376 (PMC5028791; doi:10.7717/peerj.2376)
Supplement: Supplemental Information 1 [file peerj-04-2376-s002.docx]

**Supplementary Tables and Figures:**

Supp. Table 1: Description of sample locations. Standardized locations were sampled in all 6 houses, while special locations were only sampled in a subset of the houses (due to availability of samples across houses). Water samples were collected in conical tubes, filtered, and then the filters were placed directly in the extractions. Other habitats were sampled using sterile swabs.

Supp. Table 2: Classifications of sampled extreme home environments based upon temperature, pH and chemical conditions.

Supp. Table 3: Results from PERMANOVAs testing (a) the effects of house ID on microbial composition; and (b) the effects of temperature class, pH class, and house ID on microbial composition.

(a)

| **Source** | **df** | **SS** | **MS** | **Pseudo-F** | ***P* (perm)** |
| --- | --- | --- | --- | --- | --- |
| ***House*** | ***5*** | ***6821.4*** | ***1364.3*** | ***1.8404*** | ***0.0001*** |
| Residual | 80 | 59304 | 741.3 |  | |
| Total | 85 | 66125 |  | | |

(b)

| **Source** | **df** | **SS** | **MS** | **Pseudo-F** | ***P* (perm)** |
| --- | --- | --- | --- | --- | --- |
| ***Temp Class*** | ***1*** | ***1620.5*** | ***1620.5*** | ***2.1332*** | ***0.0105*** |
| ***pH Class*** | ***1*** | ***1896.9*** | ***1896.9*** | ***3.075*** | ***0.0006*** |
| ***House*** | ***5*** | ***6551*** | ***1310.2*** | ***1.8742*** | ***0.0001*** |
| ***Temp Class x pH Class*** | ***1*** | ***3177*** | ***3177*** | ***4.8112*** | ***0.0001*** |
| Temp Class x House | 5 | 3808 | 761.59 | 1.0894 | 0.2476 |
| pH Class x House | 5 | 3071.1 | 614.23 | 0.8786 | 0.7962 |
| Temp Class x pH Class x House | 5 | 3295.4 | 659.07 | 0.94276 | 0.6311 |
| Residual | 62 | 43344 | 699.09 |  | |
| Total | 85 | 66125 |  |  |  |

Supp. Table 4: List of non-human associated microbes in extreme and non-extreme (Dunn et al. 2013) home habitats [see pdf]

Supp. Figure 1: Map of houses that were sampled for the study


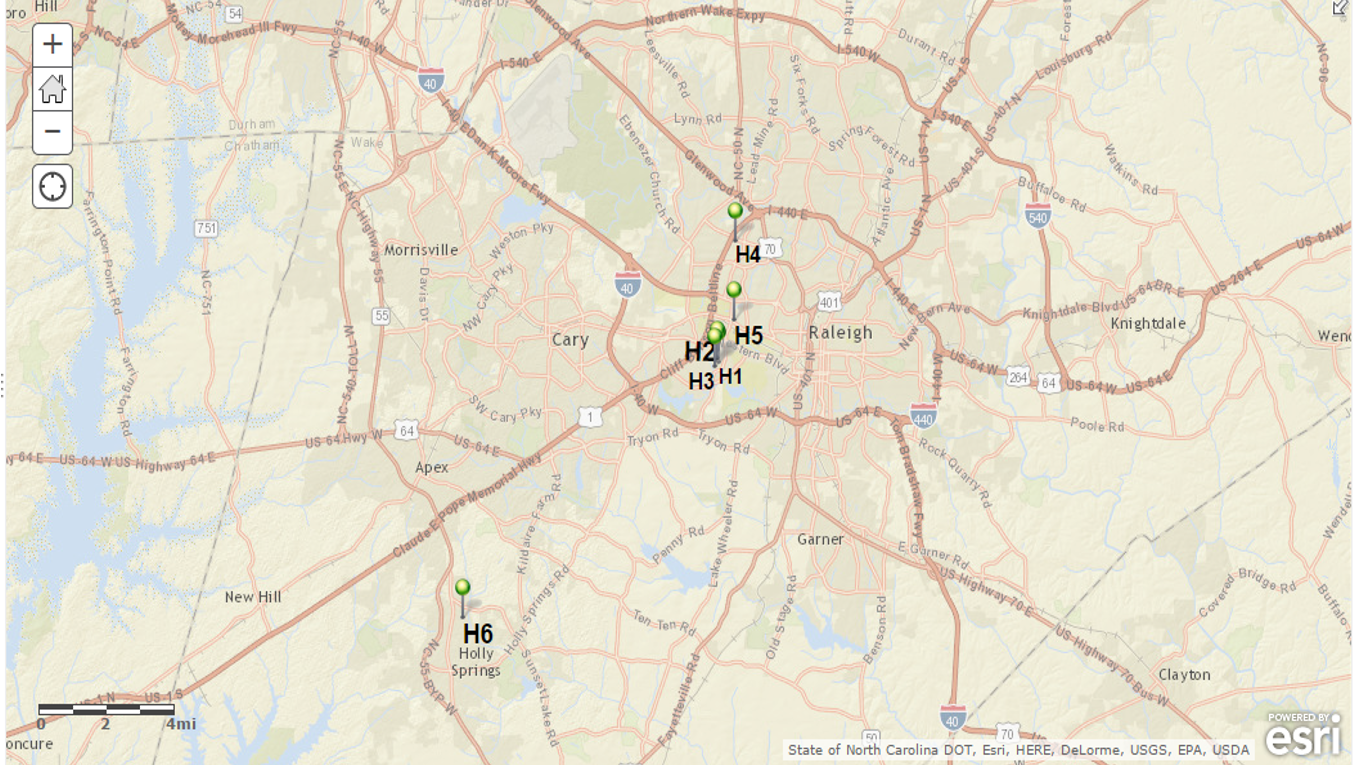


Supplemental Figure 2: NMDS plot with houses and sampling locations labeled.


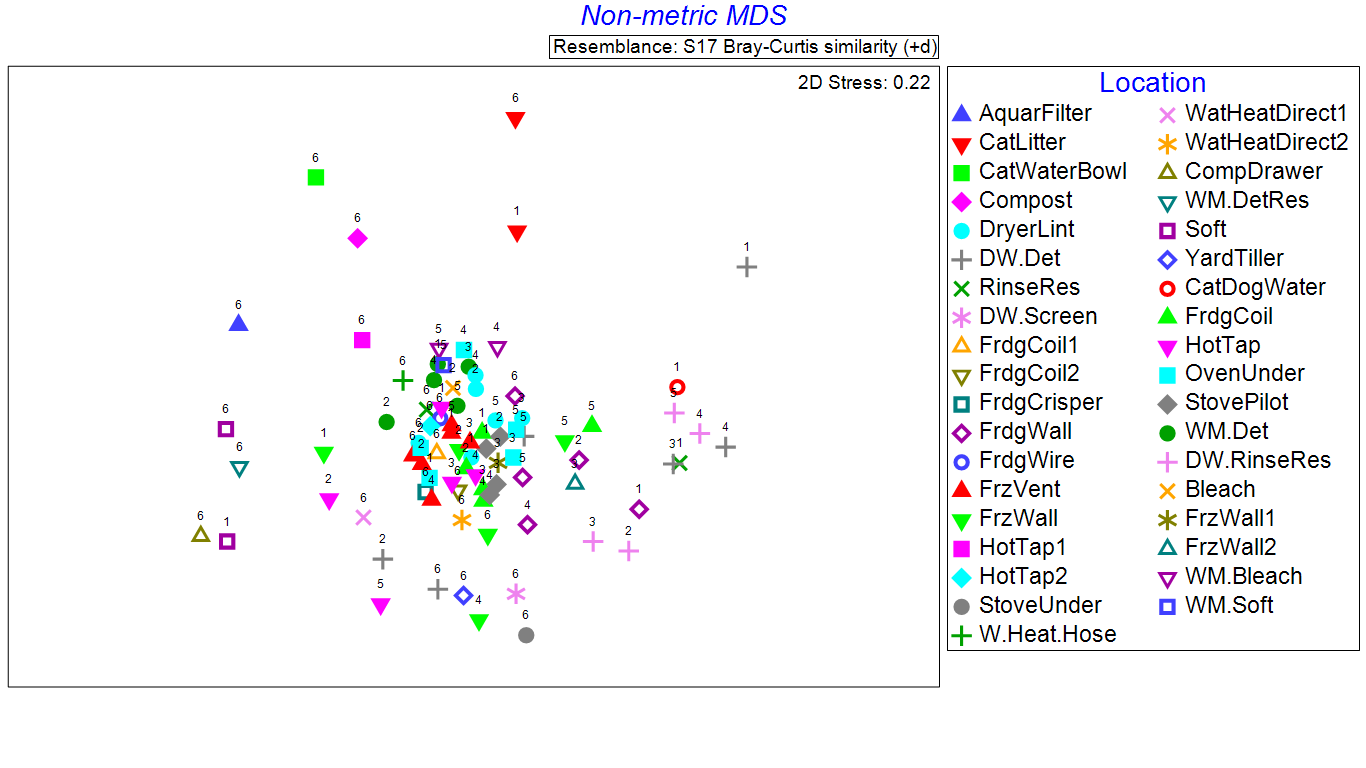


Supplemental Figure 3: Histograms depicting the % of reads from bacterial and archaeal classes in (a) habitats with extreme temperatures, (b) habitats with extreme pH, and (c) habitats with extreme chemicals.
