# Supplementary material for: Microbial diversity of extreme habitats in human homes
Source: PeerJ. 2016 Sep 13;4:e2376. doi: 10.7717/peerj.2376 (PMC5028791; doi:10.7717/peerj.2376)
Supplement: Supplemental Information 2 [file peerj-04-2376-s003.pdf]

Supp. Table 4: List of non-human associated microbes in extreme and non-extreme (Dunn et al. 2013) home

**Genus**	**Dataset_Present**
Abiotrophia	Both
Achromobacter	Both
Aerococcus	Both
Aeromicrobium	Both
Agrobacterium	Both
Alkanindiges	Both
Alloiococcus	Both
Amaricoccus	Both
Ammoniphilus	Both
Aquabacterium	Both
Balneimonas	Both
Bdellovibrio	Both
Blastomonas	Both
Bradyrhizobium	Both
Burkholderia	Both
Buttiauxella	Both
Capnocytophaga	Both
Carnobacterium	Both
Catenibacterium	Both
Caulobacter	Both
Cellulomonas	Both
Cellvibrio	Both
Chroococcidiopsis	Both
Chyrseobacterium	Both
Cohnella	Both
Comamonas	Both
Cupriavidus	Both
Devosia	Both
Dokdonella	Both
Dyadobacter	Both
Enterococcus	Both
Erwinia	Both
Exiguobacterium	Both
Facklamia	Both
Flavisolibacter	Both
Flavobacterium	Both
Flectobacillus	Both
Fusobacterium	Both
Gardnerella	Both

Gemmata	Both
Geodermatophilus	Both
Gordonia	Both
Helicobacter	Both
Hymenobacter	Both
Hyphomicrobium	Both
Janthinobacterium	Both
Jeotgalicoccus	Both
Kaistobacter	Both
Kineococcus	Both
Lautropia	Both
Leuconostoc	Both
Limnohabitans	Both
Luteibacter	Both
Megamonas	Both
Megasphaera	Both
Methylibium	Both
Methylobacterium	Both
Methylopila	Both
Methylotenera	Both
Methyloversatilis	Both
Micrococcus	Both
Microcoleus	Both
Mobiluncus	Both
Modestobacter	Both
Moraxella	Both
Nesterenkonia	Both
Nitrospira	Both
Novosphingobium	Both
Paracoccus	Both
Pediococcus	Both
Pedobacter	Both
Phenylobacterium	Both
Planctomyces	Both
Pseudoxanthomonas	Both
Psychrobacter	Both
Rheinheimera	Both
Rhizobium	Both
Rhodobacter	Both
Rhodococcus	Both

Rhodoplanes	Both
Rothia	Both
Rubellimicrobium	Both
Rubrivivax	Both
Rubrobacter	Both
Salinicoccus	Both
Schlegelella	Both
Segetibacter	Both
Sejongia	Both
Shewanella	Both
Skermanella	Both
Sphingobacterium	Both
Sphingobium	Both
Sphingomonas	Both
Sphingopyxis	Both
Spirosoma	Both
Sporosarcina	Both
Tepidimonas	Both
Thermomonas	Both
Thermus	Both
Varibaculum	Both
Wautersiella	Both
Weissella	Both
Yaniella	Both
Azobacteroides	Extreme
Azospira	Extreme
Brachybacterium	Extreme
Brevundimonas	Extreme
Brochothrix	Extreme
Buchnera	Extreme
Cetobacterium	Extreme
Elizabethkingia	Extreme
Enhydrobacter	Extreme
Fimbriimonas	Extreme
Gluconobacter	Extreme
Helcococcus	Extreme
Oligella	Extreme
Parascardovia	Extreme
Photobacterium	Extreme
Polynucleobacter	Extreme

Propionibacterium	Extreme
Pseudoalteromonas	Extreme
Ralstonia	Extreme
Salinibacterium	Extreme
Solibacter	Extreme
Thermicanus	Extreme
Xiphinematobacter	Extreme
Acetobacter	Homes
Acidaminococcus	Homes
Acidiphilium	Homes
Acidocella	Homes
Acidovorax	Homes
Actinobaculum	Homes
Actinokineospora	Homes
Actinomadura	Homes
Actinoplanes	Homes
Aggregatibacter	Homes
Agromyces	Homes
Alcaligenes	Homes
Alishewanella	Homes
Alistipes	Homes
Alkalibacterium	Homes
Alloscardovia	Homes
Amycolatopsis	Homes
Anabaena	Homes
Anaeromyxobacter	Homes
Anaerovorax	Homes
Aphanizomenon	Homes
Arcanobacterium	Homes
Arthrobacter	Homes
Arthronema	Homes
Arthrospira	Homes
Asaia	Homes
Asticcacaulis	Homes
Atopobium	Homes
Aurantimonas	Homes
Azohydromonas	Homes
Azospirillum	Homes
Bartonella	Homes
Bergeyella	Homes

Bordetella	Homes
Bosea	Homes
Brachymonas	Homes
Brenneria	Homes
Brevibacillus	Homes
Caldilinea	Homes
Calothrix	Homes
Cardiobacterium	Homes
Cellulosimicrobium	Homes
Chelativorans	Homes
Chitinophaga	Homes
Chthoniobacter	Homes
Citrobacter	Homes
Conchiformibius	Homes
Crossiella	Homes
Cryocola	Homes
Cryptosporangium	Homes
Curtobacterium	Homes
Cystobacter	Homes
Cytophaga	Homes
Deinococcus	Homes
Demequina	Homes
Dermabacter	Homes
Dermacoccus	Homes
Dermatophilus	Homes

Desemzia	Homes
Desulfomicrobium	Homes
Desulfovibrio	Homes
Dietzia	Homes
Edaphobacter	Homes
Eikenella	Homes
Ensifer	Homes
Enterobacter	Homes
Epulopiscium	Homes
Erythromicrobium	Homes
Eubacterium	Homes
Filifactor	Homes
Fluviicola	Homes
Frankia	Homes
Frateuria	Homes

Gemella	Homes
Gemmatimonas	Homes
Geobacillus	Homes
Geobacter	Homes
Georgenia	Homes
Gluconacetobacter	Homes
Granulicatella	Homes
Haloanella	Homes
Halomonas	Homes
Herpetosiphon	Homes
Hylemonella	Homes
Iamia	Homes
Ignatzschineria	Homes
Isoptericola	Homes
Janibacter	Homes
Jonquetella	Homes
Kaistella	Homes
Kaistia	Homes
Kineosporia	Homes
Kingella	Homes
Klebsiella	Homes
Knoellia	Homes
Kurthia	Homes
Kushneria	Homes
Kytococcus	Homes
Labrys	Homes
Lampropedia	Homes
Legionella	Homes
Leptolyngbya	Homes
Leptotrichia	Homes
Leucobacter	Homes
Luteimonas	Homes
Luteolibacter	Homes
Lutibacterium	Homes
Lysinibacillus	Homes
Lysobacter	Homes
Macrococcus	Homes
Marinomonas	Homes
Marmoricola	Homes
Massilia	Homes

Mesorhizobium	Homes
Methylophilus	Homes
Methylosinus	Homes
Microbacterium	Homes
Microbispora	Homes
Microcella	Homes
Microcystis	Homes
Micromonospora	Homes
Morganella	Homes
Mycoplana	Homes
Mycoplasma	Homes
Myroides	Homes
Nannocystis	Homes
Nevskia	Homes
Nocardia	Homes
Nocardioides	Homes
Nocardiopsis	Homes
Nonomuraea	Homes
Nostoc	Homes
Oceanobacillus	Homes
Oceanospirillum	Homes
Ochrobactrum	Homes
Oerskovia	Homes
Opitutus	Homes
Oribacterium	Homes
Paenibacillus	Homes
Pantoea	Homes
Patulibacter	Homes
Pedomicrobium	Homes
Phormidium	Homes
Phycicoccus	Homes
Phyllobacterium	Homes
Pigmentiphaga	Homes
Pimelobacter	Homes
Pirellula	Homes
Planomicrobium	Homes
Pontibacter	Homes
Prauserella	Homes
Prochlorococcus	Homes
Promicromonospora	Homes

Prosthecobacter	Homes
Proteus	Homes
Providencia	Homes
Pseudoclavibacter	Homes
Psychromonas	Homes
Pullulanibacillus	Homes
Ramlibacter	Homes
Renibacterium	Homes
Rhodanobacter	Homes
Rhodobaca	Homes
Rhodocyclus	Homes
Rhodoferax	Homes
Rhodopseudomonas	Homes
Rickettsia	Homes
Rickettsiella	Homes
Roseateles	Homes
Roseomonas	Homes
Rummeliibacillus	Homes
Saccharopolyspora	Homes
Sediminibacterium	Homes
Selenomonas	Homes
Serinicoccus	Homes
Shinella	Homes
Simonsiella	Homes
Sinomonas	Homes
Sneathia	Homes
Solibacillus	Homes
Solimonas	Homes
Sphaerotilus	Homes
Spiroplasma	Homes
Spongiibacter	Homes
Stenotrophomonas	Homes
Steroidobacter	Homes
Stigmatella	Homes
Tannerella	Homes
Terracoccus	Homes
Terriglobus	Homes
Tetrasphaera	Homes
Thioclava	Homes
Treponema	Homes
Tropheryma	Homes

Turicibacter	Homes
Vagococcus	Homes
Vibrio	Homes
Virgisporangium	Homes
Williamsia	Homes
Wolbachia	Homes
Wolinella	Homes
Xanthobacter	Homes
Xanthomonas	Homes
Xenophilus	Homes
Yonghaparkia	Homes

e habitats
